# Electrospun Cellulose Acetate/Poly(Vinyl Alcohol) Nanofibers Loaded with Methyl Gallate and Gallic Acid for Anti-*Staphylococcus aureus* Applications

**DOI:** 10.3390/polym16212971

**Published:** 2024-10-23

**Authors:** Pimsumon Jiamboonsri, Weradesh Sangkhun, Sompit Wanwong

**Affiliations:** 1 Faculty of Medicine, King Mongkut’s Institute of Technology Ladkrabang, 1 Chalongkrung Road, Ladkrabang, Bangkok 10520, Thailand; 2 Materials Technology Program, School of Energy, Environment and Materials, King Mongkut’s University of Technology Thonburi, 126 Pracha Uthit Road, Bang Mod, Bangkok 10140, Thailand; weradesh.s@gmail.com (W.S.); sompit.wan@kmutt.ac.th (S.W.)

**Keywords:** methyl gallate, gallic acid, cellulose acetate/poly(vinyl alcohol), electrospun nanofiber, anti-*S. aureus* material

## Abstract

Methyl gallate (MG) and gallic acid (GA) are natural compounds with potent activity against methicillin-resistant *Staphylococcus aureus* (MRSA), a significant global health concern. In this study, MG and GA were incorporated into cellulose acetate (CA) blended with poly(vinyl alcohol) (PVA) to create electrospun nanofibers aimed at combating both methicillin-susceptible *S. aureus* (MSSA) and MRSA. Key electrospinning parameters—DC voltage, injection flow rate, and syringe tip–collector distance—were optimized, with the best conditions being a 1.5 mL/h flow rate, 30 cm distance, and 20 kV voltage. The resulting nanofiber mats were characterized by SEM, FTIR, DSC, tensile strength testing, contact angle measurement, swelling behavior, and release profiling. Antibacterial properties were assessed using the agar diffusion test. The obtained nanofibers had diameters ranging from 879.33 to 906.13 nm. Among the samples, MG-GA-CA/PVA exhibited the highest tensile strength, good flexibility, and improved stiffness, which was related to enhanced thermal stability and chemical interactions as shown by DSC and FTIR analyses. This formulation also displayed excellent hydrophilicity, swelling properties, and a consistent release profile over 8 to 24 h. Furthermore, MG-GA-CA/PVA showed superior antibacterial activity against both MSSA and MRSA, suggesting its potential as a strong, flexible, and effective anti-*S. aureus* material.

## 1. Introduction

MG and GA, which are natural polyphenolic compounds abundant in many plants, foods, and beverages, have gained attention in medical and pharmaceutical research due to their wide range of pharmacological activities and safety profiles [1]. They are recognized not only as strong antioxidants [2,3,4] but also for their potent antibacterial activity [5,6]. Several studies have shown that MG and GA can inhibit and eradicate various Gram-positive and Gram-negative bacteria, particularly pathogenic antibiotic-resistant bacteria, such as *Escherichia coli* [7], *Pseudomonas aeruginosa* [8,9,10], and *S. aureus* [11]. The antibacterial mechanisms of MG and GA have been proposed to disrupt the inner/outer bacterial membrane and decrease the efflux pump gene [5,10]. Moreover, they have been reported to inhibit biofilm formation, one of key factors in bacterial resistance and virulence [12]. Sang et al. demonstrated that GA inhibits MRSA biofilm by suppressing the sarA gene [13]. Additionally, MG inhibits *P. aeruginosa* biofilm by inhibiting quorum sensing [8]. In our previous study, we demonstrated that MG and GA found in mango seed kernel extract could inhibit both susceptible and clinically resistant strains of *S. aureus* at a minimum inhibitory concentration (MIC) range of 0.19–>3 mg/mL [14]. Moreover, the addition of either MG or GA at sub-MIC levels could enhance the effectiveness of penicillin G against 19 clinical MRSA isolates by at least twofold [14]. However, our recent research showed that the synergistic mechanism of MG and GA was poorly associated with β-lactamase inhibition and was potentially related to cell barrier destruction [11]. Although MG, a methyl ester derivative of GA, has been proposed to have a similar mechanism to GA for bacterial inhibition, the combination of MG and GA showed an additive effect against both MSSA and MRSA [11]. Therefore, using MG and GA alone or in combination for antibacterial applications would not affect their potency. However, the development of MG and GA in pharmaceutical or medical fields could be limited due to their pharmacokinetic properties. GA and MG have poor oral bioavailability and are metabolized by liver cytochrome and gut bacteria [15,16]. Therefore, the development of medical materials incorporating MG and GA for antibacterial applications is intriguing for external use.

The electrospinning technique is promising for improving the physicochemical properties of fibers for healthcare applications. This technique involves using a high-voltage electric field to create charged threads of polymer solutions, forming fine fibers at the nanoscale [17]. The resulting nanofibers possess high surface-area-to-volume ratios, allowing for the incorporation of a sufficient amount of therapeutic agents [17]. Their highly porous mesh enhances breathability and fluid absorption capabilities, while their superior mechanical properties enable them to conform to the contours of the skin, ensuring better contact [18]. Additionally, they can incorporate a wide range of therapeutic agents into several polymer types [19,20,21]. These unique properties make them ideal candidates for transdermal drug delivery or biomaterial for skin [18].

Several reports demonstrate the successful use of electrospun nanofibers for antibacterial applications by incorporating various active compounds, including therapeutic agents, herbal extracts, and active chemical substances from natural sources. For example, vancomycin, a first-line agent for treating MRSA infections, loaded into poly(ethylene oxide) and sodium alginate blended nanofibers, showed superior efficiency in inhibiting MRSA in both in vitro and animal models compared to the free drug [22]. Fahimirad et al. demonstrated that plant extract from *Echinacea purpurea* incorporated into polycaprolactone/PVA-chitosan lactate-based electrospun nanofibers achieved 100% MRSA growth suppression in a colony count assay [23]. In case of plant bioactive compounds, rutin loaded in cellulose acetate (CA) blended with poly(ethylene oxide) nanofiber mats could inhibit both *E. coli* and *S. aureus* greater than 93% [24]. As aforementioned, various polymers are used to fabricate electrospun nanofibers for antibacterial applications. Among biodegradable and biocompatible polymers, CA and PVA are of particular interest due to their unique properties. PVA is a synthetic water-soluble polymer that provides good mechanical strength and flexibility [25], while CA is a semi-synthetic water-insoluble polymer with excellent film-forming properties and solvent resistance [26]. Therefore, combining CA and PVA to fabricate nanofibers offers the advantages of versatile active compound loading and tailored release profiles, allowing for the customization to meet specific application requirements.

In this work, we aimed to develop CA/PVA nanofibers loaded with GA and MG for anti-MRSA applications. We evaluated the basic parameters of the electrospinning technique, including voltage, injection flow rate, and the distance between the injection tip and collector. The resulting nanofibers were characterized by using various techniques such as a scanning electron microscope (SEM), Fourier transform infrared spectroscopy (FTIR), and differential scanning calorimeter (DSC) analysis, as well as tensile stress, water contact angle, and drug release measurements. Finally, the antibacterial properties were assessed against both MSSA and MRSA strains.

## 2. Materials and Methods

### 2.1. Materials

GA (98.8%) was purchased from EMD Millipore (Buchs, Switzerland). MG (>98%) and PVA (*n* = approx. 1700) were obtained from Tokyo Chemical Industry (Tokyo, Japan). Cellulose acetate (CA) (Mn = 30,000) was purchased from Sigma Aldrich (St. Louis, MO, USA). Acetic acid (glacial) was sourced from Merck (Darmstadt, Germany).

### 2.2. Electrospinning of CA/PVA Nanofibers

The stock solutions of 10 wt% of CA and PVA in solution were prepared by mixing 0.65 g of CA and 5.85 g of PVA into a solution of acetic acid (40 mL) in DI water (25 mL). The solution was then stirred and heated at 85 °C for at least 1 h until fully dissolved. For the electrospinning process, 5 mL of the degassed solution was transferred into a 10 mL plastic syringe fitted with an 18 G blunt-tip syringe needle. The injection angle was positioned horizontally at an angle of 0°. The electrospinning process was conducted under atmospheric conditions with a humidity of 75%RH. The effects of three parameters, including applied DC voltage, injection flow rate, and the distance between the syringe tip and the aluminum collector, were systematically evaluated.

To prepare CA/PVA nanofibers loaded with active compounds, the amounts of MG and GA were calculated based on our previous study [11]. For MG-CA/PVA and GA-CA/PVA nanofibers, 75.52 mg of MG (7.5 wt% of polymer) or 9.44 mg of GA (0.9 wt% of polymer) was added into 10 mL of CA/PVA solution to prepare the spinning solution. For the MG-GA-CA/PVA solution, similar amounts of MG and GA were added into 10 mL of the CA/PVA solution. The concentrations for each formulation are summarized in Table 1. The solution was then ultrasonicated for 20 min before fabricating nanofibers using the optimized condition. The obtained nanofibers were dried in a vacuum oven at 60 °C and 10 mbar overnight to remove the residue solvents.

### 2.3. Characterizations

#### 2.3.1. Physical, Chemical, Thermal, and Mechanical Properties

The structural characteristics of MG, GA, and MG-GA loaded CA/PVA nanofibers were investigated using a field-emission SEM (Versa 3D FEG). The chemical modification of nanofibers was analyzed using an FTIR Thermo Scientific, Nicolet 6700 spectrophotometer. The FTIR spectra were recorded over the range of 700–4000 cm^−1^. The thermal properties of the samples were measured by a DSC (NETZSCH DSC 204 F1 Phoenix) where the samples were heated from 50 °C to 250 °C with a rate of 5 °C/min. Mechanical properties, including tensile strength, Young’s modulus, and strain at break of the nanofiber mats, were determined by using a Universal Testing Machine (Instron^®^, model 55R4502, Norwood, MA, USA) following sample preparation and testing conditions according to ASTM D638-10 standards [27].

#### 2.3.2. Contact Angle Analysis

The hydrophilic and hydrophobic natures of the CA/PVA, MG-CA/PVA, GA-CA/PVA and MG-GA-CA/PVA mats were determined by measuring water contact angles. Three different zones from each sample, sized 3 × 3 cm^2^, were analyzed using an optical contact angle meter (KINO Scientific Instrument Inc., model SL150E, Somerville, MA, USA).

#### 2.3.3. Swelling Test

The swelling test of electrospun fiber was performed by immersing electrospun fiber (1.5 × 1.5 cm^2^) in DI water for 24 h. Before the test, the dry weight of electrospun fiber was measured. After immersion, the excess surface water on the swollen fiber was removed using filter paper, and the fiber was then dried at room temperature for 24 h. The weight of the swollen fiber was subsequently measured. Three samples per formulation were tested. The swelling ratio was determined using the Equation (1):

(1) $$Degree\;of\;Swelling\left(\%\right)=\frac{W_{s}-W_{0}}{W_{0}}\times 100$$

where W_s_ and W_0_ are the weights of the swollen fiber and the dried fiber, respectively.

#### 2.3.4. Release Test

The specimens (1.5 × 1.5 cm^2^, approximately 40 mg) were immersed in 10 mL of DI water for 24 h. Samples were collected at 8 and 24 h. The solution was then filtered using a 0.45 µm hydrophilic PTFE syringe filter, and the released concentration was measured using a UV-visible spectrophotometer (Thermo Scientific, GENESYS 10S, Waltham, MA, USA) at wavelength 260 nm for GA and 290 nm for MG. The linear regression equation for MG and GA in the concentration range of 0.02–0.45 mM was Y = 6.7528X − 0.1019 for MG and Y = 6.7518X − 0.2105 for GA with an r^2^ of 0.9999. The results were reported as the amount of MG or GA released per gram of sample.

### 2.4. Anti-S. aureus Activity Test

*S. aureus* ATCC 25923 (MSSA) and ATCC 43300 (MRSA) were purchased from the American Type Culture Collection. The bacteria were maintained in a mixture of tryptic soy broth (TSB; Becton Dickinson & Co., Cockeysville, MD, USA) and 20% *w*/*v* glycerol at −80 °C until use. For experiments, both bacterial strains were grown separately on Muller Hilton broth (Becton Dickinson & Co., Cockeysville, MD, USA) at 37 °C for 24 h. Subsequently, the bacterial suspension was adjusted for turbidity by using a UV-visible spectrophotometer (Thermo scientific, GENESYS 10S) at 600 nm to obtain an optical density (OD) of 0.1, which contained approximately 10^7^ colony-forming units (CFU/mL). All test samples were cut into a size of 1 × 1 cm^2^ and pre-sterilized by ultraviolet light for 30 min before the test.

The antibacterial activities of the nanofiber mats were tested by the agar diffusion method. Briefly, each prepared bacterial suspension was swabbed on Mueller-Hinton agar (Becton Dickinson &Co., Cockeysville, MD, USA) and air–dried at room temperature (25 °C). The pre-sterile mats were placed on the agar plate. After incubation at 37 °C for 24 h, the inhibition diameters (mm) were measured, and the experiments were performed in triplicate. Sterile paper filter with a size of 1 × 1 cm^2^ loaded with ampicillin (10 μg/cm^2^) was used as the antibiotic control, and the CA/PVA mat was used as the negative control.

### 2.5. Statistical Analysis

The statistical analyses were performed using SPSS (version 29.0, SPSS Inc., Chicago, IL, USA). An analysis of variance (ANOVA) was performed, and significant differences between means were determined using Tukey’s honesty significant difference test or Dunnett’s T3 test at a significance level of *p* < 0.05.

## 3. Results and Discussion

### 3.1. Optimization of Electrospinning Conditions

**Figure 1:**
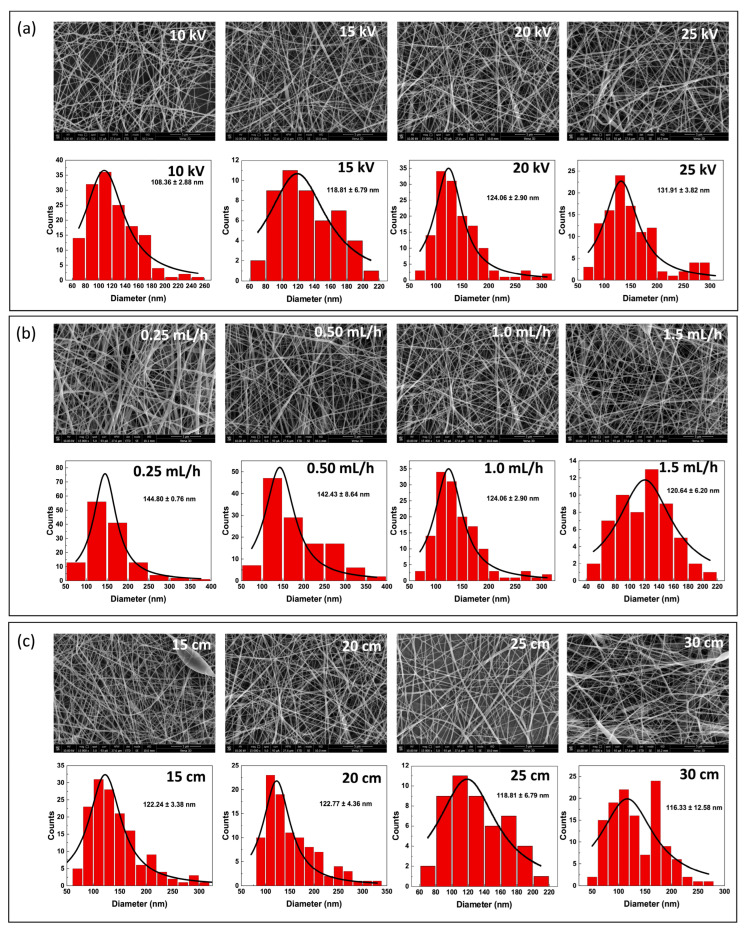
(**a**) The effect of the applied DC voltages (10, 15, 20, and 25 kV) at a distance of 20 cm and a flow rate of 1 mL/h, (**b**) the effect of injection flow rates (0.25, 0.50, 1.00, and 1.50 mL/h) at a distance of 20 cm and a voltage of 20 kV, and (**c**) the effect of the distance between the syringe tip and the collector (15, 20, 25, and 30 cm) at a flow rate of 1.5 mL/h and a voltage of 20 kV on the morphology of nanofibers, shown through SEM images at magnification of 15,000× and corresponding size diameter distribution (red histograms).

Three important factors—applied voltage, injection flow rate, and the syringe-tip-to-collector distance—were optimized to fabricate CA/PVA nanofiber mats without active substances. First, the influence of applied DC voltages on the size and morphology of the nanofibers was investigated at a fixed flow rate of 1 mL/h and a distance of 20 cm. As shown in Figure 1a, the average diameter of the obtained fibers ranged from 108 ± 2.88 to 131.91 ± 3.82 nm as the applied DC voltage increased from 10 to 25 kV. However, the nanofibers produced at low voltages (10 and 15 kV) presented a smooth surface with more visible beads compared to those produced at higher voltages (20 and 25 kV). Additionally, diameter histograms at 20 kV showed a sharper distribution peak than those at 25 kV, suggesting that most of fiber counts fell within a narrow diameter range. Therefore, to optimize the flow rate in this study, the voltage was fixed at 20 kV with a distance of 20 cm.

According to the literature, increasing the voltage can have two opposing effects on fiber diameter during electrospinning [28,29]. Higher voltage increases the electric field strength, pulling the polymer solution more forcefully and resulting in thinner fibers [28]. In contrast, higher voltage can also lead to larger fibers due to an increase in jet velocity, which causes a greater volume of material to be ejected [28]. Teixeira et al. demonstrated that the diameter of 100% PVA electrospun nanofibers increased with increasing voltage, whereas the diameter of 90/10 blended PVA/CA electrospun nanofibers increased with decreasing voltage [30]. Additionally, parameters related to the polymer solution may have a more significant effect on the nanofiber sizes and morphologies rather than the parameters related to the electrospinning process. Angel et al. reported that altering the voltage and nozzle–collector distance did not consistently affect the CA fiber diameter which was significantly influenced by polymer concentrations [29]. By using a fixed concentration of CA/PVA in our study, the parameters related to the electrospinning process became the primary factors affecting fiber sizes and morphology.

In Figure 1b, the mean diameters of CA/PVA fibers decreased from 144.80 ± 0.76 to 120.64 ± 6.20 nm as the flow rate increased from 0.25 to 1.5 mL/h. At the highest flow rate of 1.5 mL/h, the mean diameter histogram demonstrated a wide normal distribution in the range of 40–220 nm, while the mean diameters observed at 0.25, 0.5, and 1 mL/h displayed right-skewed distributions in the range of 50–400 nm. Additionally, no visible beads were observed under any conditions, as illustrated in SEM micrographs in Figure 1b. Typically, large fiber diameters with beads form when the flow rate increases because a greater volume of material is drawn from the nozzle tip, and the solvent has inadequate time to evaporate [31]. However, when the flow rate exceeds a certain threshold, a decrease in fiber diameter can occur. Schoenmaker et al. demonstrated that polyamide fiber diameters increased with increasing flow rate from 2 to 4.5 mL/h, but decreased when the flow rate exceeded 4.5 mL/h due to the increasing amount of charges [32]. In this study, the difference in mean diameter size observed at 1 mL/h and 1.5 mL/h was very small. This suggests that beyond a certain point, increasing the flow rate may not significantly reduce the fiber size any further. Therefore, balancing flow rate with other parameters is necessary under specific electrospinning conditions. The distance between the syringe tip and the collector is an important parameter to optimize. When the distance is either too short or too long, it affects the fiber size and bead formation. Thus, the influence of distance was studied at a fixed voltage of 20 kV and a flow rate of 1.5 mL/h. As the distance increased from 15 to 30 cm, the diameter of the nanofibers decreased from 122.24 ± 3.38 to 116.33 ± 12.58 nm. The SEM micrographs showed no difference in fiber morphology. This result aligns well with the understanding that longer distances allow the solvent to vaporize before reaching the collector, forming thinner fibers [33].

Therefore, the mean fiber diameter in our electrospinning condition decreased with decreasing applied voltage, increasing injection flow rate, and increasing the syringe tip–collector distance (Figure 1). Based on these optimization results, the spinning conditions with a flow rate of 1.5 mL/h, an applied voltage of 20 kV, and a distance of 30 cm were further used to fabricate nanofiber loaded with MG and GA.

### 3.2. Characterization of Electrospun Fiber Mats Loaded with Phenolic Compounds

#### 3.2.1. SEM

**Figure 2:**
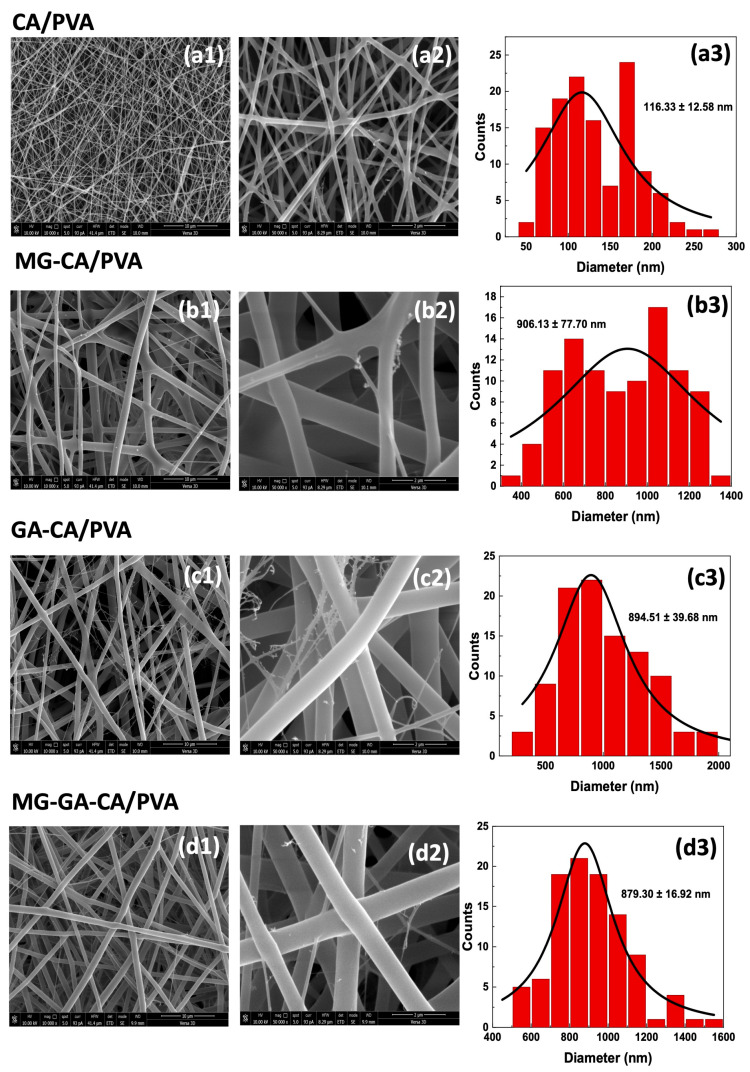
SEM images of (**a1**,**a2**) CA/PVA, (**b1**,**b2**) MG-CA/PVA, (**c1**,**c2**) GA-CA/PVA, and (**d1**,**d2**) MG-GA-CA/PVA nanofiber patches at magnifications of 10,000× and 50,000× each, along with their fiber diameter distributions ((**a3**–**d3**), red histograms).

Figure 2 demonstrates the morphology of nanofibers and the distributions of fiber diameter for CA/PVA loaded with active compounds. The results showed that the average diameter of the nanofibers significantly increased after the loading of phenolic compounds. The average diameters of CA/PVA loaded with either GA or MG increased by more than 7.5-fold (894.51 ± 39.68 and 906.13 ± 77.70 nm, respectively) compared to the neat CA/PVA nanofiber (116.33 ± 12.58 nm). This result is consistent with a previous report that the addition of 10 to 40 wt% GA into a CA solution could increase nanofiber diameters from approximately 540 to 786 nm, where the diameter of neat CA nanofibers was about 335 nm [34]. Similarly, the size of PVA electrospun fibers increased with the addition of GA [35]. The increase in the diameter of nanofibers when adding active content may be due to the increased viscosity of the polymer solution [34].

#### 3.2.2. FTIR

**Figure 3:**
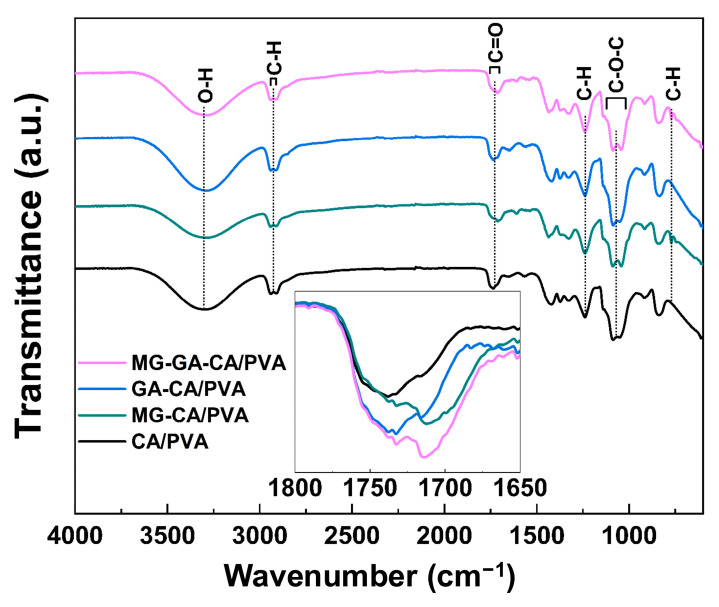
FTIR spectra of CA/PVA, MG-CA/PVA, GA-CA/PVA, and MG-GA-CA/PVA nanofibers.

The FTIR spectra of all electrospun nanofibers are depicted in Figure 3. The CA/PVA, MG-CA/PVA, GA-CA/PVA, and MG-GA-CA/PVA nanofibers exhibited a broad absorption band at 3308 cm^−1^, corresponding to the O-H stretching vibration. The introduction of MG, GA, and MG-GA combination into the CA/PVA matrix led to an increased peak intensity compared to the baseline spectrum of CA/PVA, indicating enhanced chemical interactions within the matrix, particularly involving the hydroxyl groups of phenolic compounds and PVA.

Among the CA/PVA nanofibers, the MG-GA-CA/PVA composite spectrum showed the most pronounced changes, with stronger peaks, as highlighted in the magnified spectra inset in Figure 3. The CA/PVA and GA-CA/PVA nanofibers exhibited a peak at 1740 cm^−1^, attributed to the stretching vibration of the carbonyl (–C=O) group present in CA and GA-CA [36]. This suggests that GA interacts with the CA matrix.

In contrast, the MG-CA/PVA and MG-GA-CA/PVA nanofibers displayed additional peaks at 1715 cm^−1^, which is attributed to the C=O stretching of the ester group in the MG compound [37]. The strong peaks observed at 1130 and 1040 cm^−1^ correspond to the asymmetric and symmetric stretching of the C–O–C bond, characteristic of ester linkages [38]. These results confirm the molecular interactions between the phenolic compounds and the CA/PVA fibers, indicating the stability of MG and GA in the matrix.

#### 3.2.3. DSC

**Figure 4:**
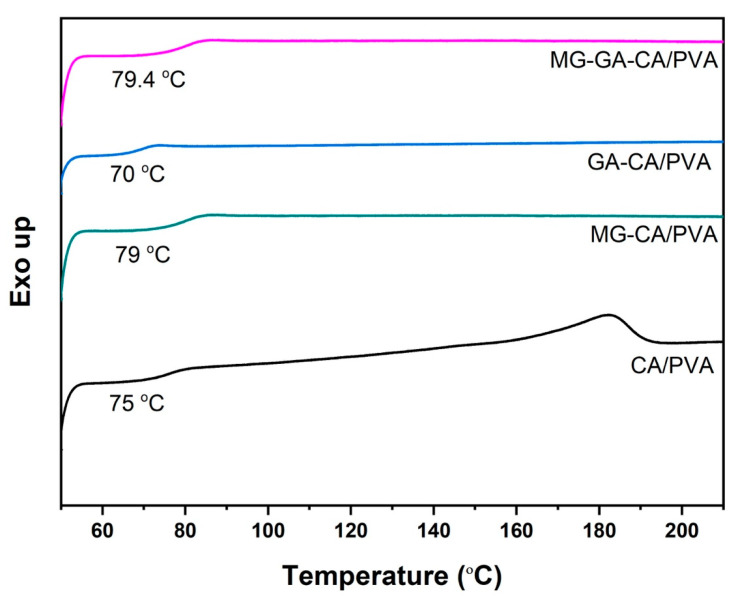
DSC thermogram of CA/PVA, MG-CA/PVA, GA-CA/PVA, and MG-GA-CA/PVA nanofibers (heating rate 5 °C/min).

The DSC thermogram in Figure 4 demonstrated endothermic peaks corresponding to the melting temperatures of nanofibers. The CA/PVA nanofiber exhibited T_g_ of 75 °C, which is in a typical range for T_g_ values of PVA (75–85 °C) [39]. The increased T_g_ values of MG-CA/PVA (79 °C) and MG-GA-CA/PVA (80 °C) indicate that the thermal stability and rigidity of nanofibers were slightly increased. This is due to hydroxyl group of MG, which can form intermolecular H-bonding with the CA/PVA matrix [40,41]. In contrast, GA-CA/PVA showed a slight decrease in T_g_ to 70 °C. This result suggests that GA may slightly disrupt the ordered structure within the nanofiber, leading to a reduction in thermal stability.

### 3.3. Tensile Strength

**Figure 5:**
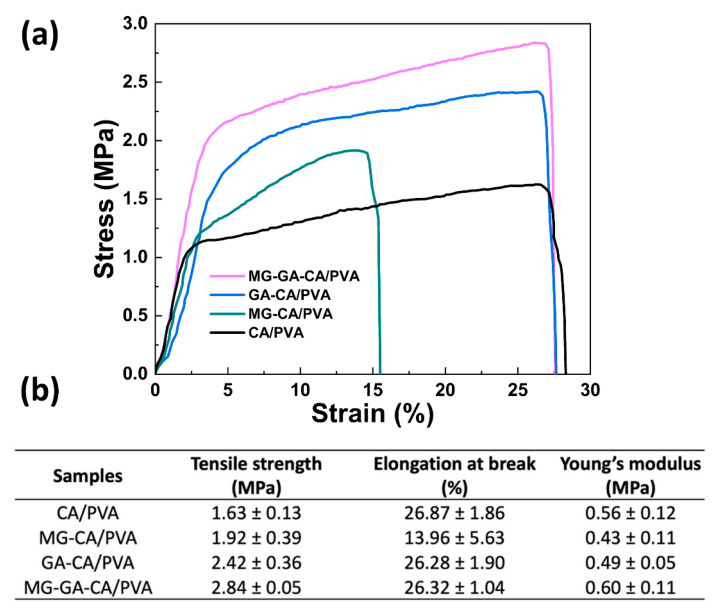
(**a**) Stress and strain curves and (**b**) summarized mechanical properties of CA/PVA, MG-CA/PVA, GA-CA/PVA, and MG-GA-CA/PVA nanofibers. Data shown as mean ± S.D. (*n* = 5).

The mechanical properties of CA/PVA nanofibers were characterized by tensile strength measurements, providing specific values for tensile strength, elongation at break, and Young’s modulus. As displayed in the stress-strain curves of Figure 5a, the addition of active phenolic compounds enhances the tensile strength of the CA/PVA nanofiber. The highest tensile strength was obtained from the MG-GA-CA/PVA nanofiber, followed by the GA-CA/PVA and MG-CA/PVA, respectively. These results could be explained by the increasing T_g_, which often correlates with the improved tensile strength. Along with the FTIR spectra, the hydrogen bonding between MG-GA and the CA/PVA matrix enhances intermolecular bonding, leading to a more stronger material [42].

As shown in Figure 5b, the elongation at break significantly decreases from 26.87 ± 1.86% to 13.96 ± 5.63% with the MG modification, indicating increased brittleness. However, the elongation at break of GA-CA/PVA and MG-GA-CA/PVA are not significantly different from the neat CA/PVA fiber. These results suggest that the addition of GA restored the elongation capacity, and the addition of the MG-GA combination could maintain this flexibility.

The Young’s modulus measures the stiffness of the material. As displayed in Figure 5b, although the variations in Young’s modulus are relatively small, the order of fiber stiffness was as follows: MG-GA-CA/PVA > CA/PVA > GA-CA/PVA > MG-CA/PVA. It could be noted that the addition of a single phenolic compound slightly decreases stiffness, while the MG-GA combination provided a slight increase modulus, suggesting a mild reinforcement effect.

These results indicated that the mechanical behaviors of the CA/PVA nanofiber mat were improved by incorporating MG-GA. A similar finding was reported by Luo et al., which indicated that incorporating tea polyphenol in a PVA nanofiber increased the tensile strength and elongation at break [43].

### 3.4. Contact Angle

**Figure 6:**
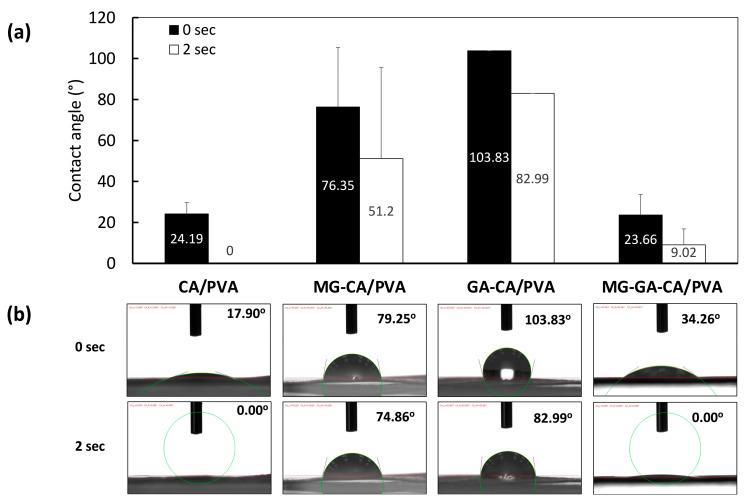
(**a**) Average water contact angle on CA/PVA, MG-CA/PVA, GA-CA/PVA, and MG-GA-CA/PVA nanofiber mats and (**b**) the corresponding representative images of water droplets at 0 and 2 s from zone 2. Data shown as mean ± S.D. (*n* = 3).

The hydrophilicity of the CA/PVA nanofiber mat loaded with phenolic compounds was assessed by measuring the water contact angle (Figure 6). Theoretically, a contact angle of less than 90° is considered as a hydrophilic surface, while more than 90° demonstrates a hydrophobic surface [44]. The results showed that the CA/PVA electrospun mat exhibited a hydrophilic surface with an initial contact angle of water of 24.19 ± 5.47°, and the water drop on the mat was completely absorbed after 2 s. However, the addition of the phenolic compound into the CA/PVA mat changed the surface to become more hydrophobic. At the initial time, the contact angle of the MG-CA/PVA fiber was 76.35 ± 29.04°, which was comparable to that of GA-CA/PVA (103.83 ± 0.00°). After a water drop, the contact angles for both mats were less than 90° within 2 s, indicating the spreading of the liquid on the solid surface. Interestingly, the addition of both GA and MG into the CA/PVA nanofibers decreased the contact angle by at least 2-fold compared with either MG-CA/PVA or GA-CA/PVA. The average water contact angle of MG-GA-CA/PVA was 23.66 ± 9.97° at the initial time (Figure 6a), and then a water drop was almost completely absorbed after 2 s (Figure 6b).

It is noteworthy that the molecular structures of MG and GA are phenolic, each containing three hydroxyl groups. As evidenced by FTIR results, hydrogen bonding between phenolic compounds and CA/PVA chains could reduce the polarity of the matrix. Consequently, the decreased availability of hydroxy groups to interact with water molecules leads to an increased hydrophobicity of the nanofiber mat. Although MG was added to the polymer solution in a higher amount than GA, the comparable contact angles between MG-CA/PVA and GA-CA/PVA might be balanced by the higher polarity of GA due to its carboxyl group. Moreover, the addition of both phenolic compounds resulting in the similar hydrophilicity and wettability characteristics between MG-GA-CA/PVA and the blank CA/PVA may be attributed to the increased availability of hydroxyl groups from phenolic compounds. This phenomenon is similar to findings in other research on PVA films with phenolic compounds [45,46,47].

### 3.5. Swelling Behavior

**Figure 7:**
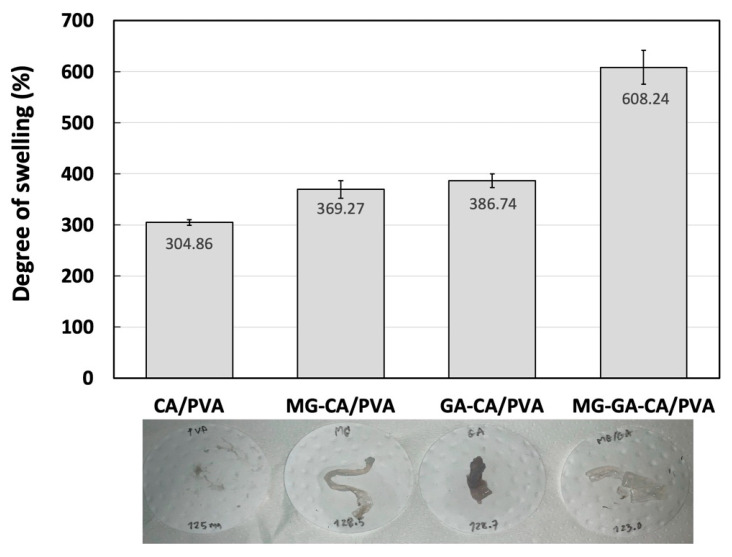
Degree of swelling of CA/PVA, MG-CA/PVA, GA-CA/PVA, and MG-GA-CA/PVA nanofiber mats and their corresponding figures. Data shown as mean ± S.D. (*n* = 3).

The swelling percentage of the neat CA/PVA nanofiber mats in DI water after 24 h was 304.86 ± 5.62%. When loaded with one or two active substances, the swelling percentage increased by at least 21.12% and 99.51%, respectively (Figure 7). Generally, smaller fiber diameters result in a larger surface-area-to-volume or mass ratio of the fiber mat, which could explain higher water retention and increased swelling percentage [34]. However, the results of this study showed that MG-GA-CA/PVA, which exhibited the largest fiber diameter, also had the highest swelling percentage, followed by GA-CA/PVA and MG-CA/PVA. It is important to note that water uptake ability mainly depends on the hydrophilic or hydrophobic nature of the materials. Considering the contact angle properties, the MG-GA-CA/PVA nanofiber showed the most hydrophilic surface, becoming completely wet within 2 s, compared to fibers loaded with MG or GA alone. Therefore, the more hydrophilic surface of MG-GA-CA/PVA likely contributes to its high-water absorption ability, leading to an increased swelling percentage. Although the MG-GA-CA/PVA nanofiber mat exhibited a similar contact angle to the neat CA/PVA mat, the CA/PVA mat showed the lowest degree of swelling, as calculated based on weight loss. Since PVA is a hydrophilic, water-soluble polymer, PVA nanofibers rapidly dissolve in water, leading to significant weight loss [48]. However, the stability of PVA-based nanofibers in water can be improved through chemical bonding with other substances [49]. Based on the FTIR results, the swelling test results suggest that the increased swelling degree of the MG-GA-CA/PVA nanofiber mat may also be due to the intramolecular bonding between the phenolic compounds and PVA/CA.

### 3.6. Release Behavior

**Figure 8:**
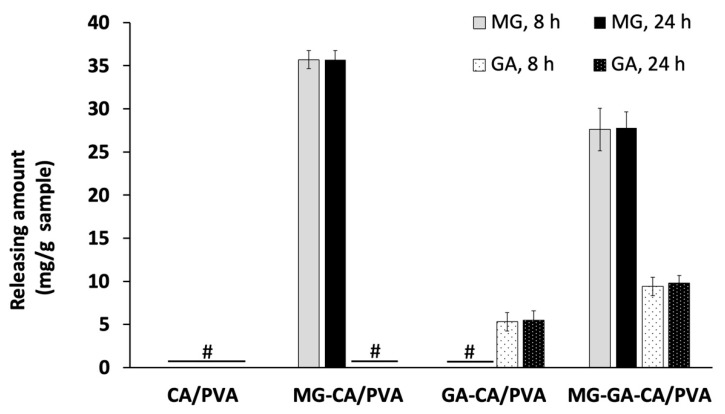
Cumulative release of MG and GA at 8 and 24 h from CA/PVA electrospun. # not detected.

The release of MG and GA from the nanofiber mat into DI medium was measured at 8 and 24 h. As shown in Figure 8, the amounts of both MG and GA released from each formulation of the CA/PVA samples at 8 h were not significantly different (*p* > 0.05) from those released at 24 h. This result suggests that the release of MG and GA from the CA/PVA mat may reach saturation by 8 h and sustained until 24 h. Approximately 35.7 mg/g of MG was released from the MG-CA/PVA sample, while approximately 5.3 mg/g of GA was released from the GA-CA/PVA sample. The released MG and GA from the single compound loaded in CA/PVA were approximately 47.3% and 56.14%, respectively, based on the dry weight of the neat electrospun material.

The release behavior of MG or GA was consistent with previous studies on phenolic compounds. Chuysinuan et al. demonstrated that the release of GA from 2.5% to 7.5% GA/PVA electrospun fibers exhibited a burst release within the first 60 min, followed by a plateau phase before 8 h [35]. Similarly, the initial burst release of GA from CA nanofibers was observed, followed by a gradual increase until reaching a plateau phase [34].

For the MG-GA-CA/PVA sample, the release of GA increased by approximately 1.7-fold compared to that from the GA-CA/PVA sample, while the release of MG decreased by approximately 1.3-fold compared to that from the MG-CA/PVA sample. Since MG is the methyl ester of GA, its hydrolysis under acidic or alkaline conditions, as well as enzymatic metabolism, results in GA as the major product [11]. Therefore, the increased release of GA and decreased release of MG from the MG-GA-CA/PVA sample may be attributed to the degradation of MG after its release into the medium.

### 3.7. Anti-S. aureus Activity

**Figure 9:**
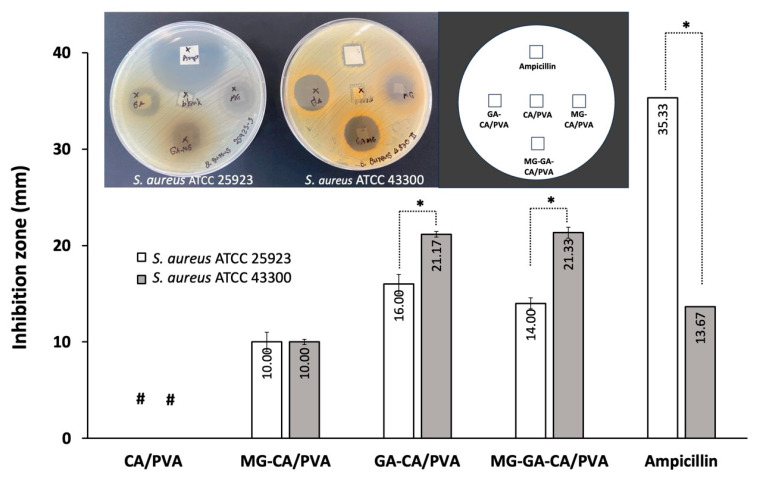
The antibacterial activities of MG-CA/PVA, GA-CA/PVA, and MG-GA-CA/PVA compared with neat CA/PVA and ampicillin against *S. aureus* ATCC 25923 (MSSA) and 43300 (MRSA). Each symbol indicates the mean ± S.D. (*n* = 3). # The inhibition zone could not be determined. * Significant difference, *p* < 0.05.

The antibacterial activities of MG-CA/PVA, GA-CA/PVA, and MG-GA-CA/PVA were demonstrated in Figure 9. It was observed that all fabricated electrospun mats were shrunk and swollen on the agar plates due to their hydrophilic and wetting ability. In contrast, the reference ampicillin, which was applied on filter paper, maintained the shape of the substrate. Consequently, the size of the fabricated electrospun mats visibly changed after incubation on the agar plate for 24 h. The reference control, ampicillin, exhibited inhibition zones of 35.33 ± 0.58 mm against *S. aureus* ATCC 25923 and 13.67 ± 0.58 mm against *S. aureus* ATCC 43300. The smaller inhibition zone observed for *S. aureus* ATCC 43300 confirms that this strain is resistant to β-lactam antibiotics and is less susceptible to them. For the neat CA/PVA mat, a light density of bacterial growth was observed underneath the sample, with no clear inhibition zone around it. This result suggests that the 10 wt% CA/PVA nanofiber lacks antibacterial activity. The result in this study was in accordance with Youdhestar et al. in which neat CA (18 wt%) and neat PVA (10 wt%) nanofibers did not exhibit antibacterial activity [50]. Among the CA/PVA nanofiber mats, MG-CA/PVA showed the smallest clear zone with an average diameter of approximately 10 mm against both MSSA and MRSA. In addition, CA/PVA loaded with GA and MG-GA demonstrated the highest inhibition zone against MRSA. GA-CA/PVA and MG-GA-CA/PVA exhibited larger clear zones against MRSA than MSSA. Unsurprisingly, these results align with our previous study, which found that GA was more effective against MRSA than MSSA in a disc diffusion assay [11]. Additionally, no significant antibacterial interaction between GA and MG was observed for each bacterial strain [11]. These findings highlight the potential of GA-CA/PVA and MG-GA-CA/PVA nanofibers to control MRSA.

## 4. Conclusions

MG (7.5 wt%) and GA (0.9 wt%) were successfully incorporated into 10 wt% of the CA/PVA nanofibers via electrospinning, using optimized conditions: 1.5 mL/h flow rate, 20 kV applied voltage, and 30 cm distance. The resulting nanofibers were bead-free, with FTIR confirming molecular interactions between the active compounds and the CA/PVA matrix. Among the samples, MG-GA-CA/PVA exhibited the highest tensile strength, good flexibility, and slightly improved stiffness, making it suitable for strong and flexible nanofiber composites. It also showed excellent hydrophilicity, swelling properties, and complete release within 24 h. Furthermore, MG-GA-CA/PVA demonstrated the best antibacterial effectiveness against both MSSA and MRSA strains. This study provides valuable insights into the fabrication of phenolic compound-loaded CA/PVA nanofibers for anti-*S. aureus* applications. However, the research was limited to one in vitro functional activity, which may not be directly applicable to the medical field. Further studies on in vitro cytotoxicity, in vivo antibacterial activity, and the stability of nanofibers are necessary to ensure their medical relevance.

## Figures and Tables

**Table 1:** Concentration of each compound used in the formulations.

**Compounds**	**Concentrations (mg/mL)**		
	**MG-CA/PVA**	**GA-CA/PVA**	**MG-GA-CA/PVA**
PVA	90	90	90
CA	10	10	10
MG	7.5	-	7.5
GA	-	0.9	0.9

## Data Availability

The data are contained within the article.
